# Ligamentum Flavum Hypertrophy in Asymptomatic and Chronic Low Back Pain Subjects

**DOI:** 10.1371/journal.pone.0128321

**Published:** 2015-05-26

**Authors:** Justin J. Munns, Joe Y. B. Lee, Alejandro A. Espinoza Orías, Ryota Takatori, Gunnar B. J. Andersson, Howard S. An, Nozomu Inoue

**Affiliations:** Department of Orthopedic Surgery, Rush University Medical Center, Chicago, Illinois, United States of America; Affiliated Hospital of North Sichuan Medical College, CHINA

## Abstract

**Purpose:**

To examine ligamentum flavum thickness using magnetic resonance (MR) images to evaluate its association with low back pain symptoms, age, gender, lumbar level, and disc characteristics.

**Materials and Methods:**

Sixty-three individuals were part of this IRB-approved study: twenty-seven with chronic low back pain, and thirty-six as asymptomatic. All patients underwent MR imaging and computed tomography (CT) of the lumbar spine. The MR images at the mid-disc level were captured and enlarged 800% using a bilinear interpolation size conversion algorithm that allowed for enhanced image quality. Ligamentum flavum thickness was assessed using bilateral medial and lateral measurements. Disc height at each level was measured by the least-distance measurement method in three-dimensional models created by CT images taken of the same subject. Analysis of variance and *t*-tests were carried out to evaluate the relationship between ligamentum flavum thickness and patient variables.

**Results:**

Ligamentum flavum thickness was found to significantly increase with older age, lower lumbar level, and chronic low back pain (*p* < 0.03). No difference in ligamentum flavum thickness was observed between right and left sided measurements, or between male and female subjects. Disc height and both ligamentum flavum thickness measurements showed low to moderate correlations that reached significance (*p* < 0.01). Additionally, a moderate and significant correlation between disc degeneration grade and ligamentum flavum thickness does exist (p <0.001).

**Conclusion:**

By measuring ligamentum flavum thickness on MR images at two different sites and comparing degrees of disc degeneration, we found that ligamentum flavum thickness may be closely related to the pathogenesis of pain processes in the spine.

## Introduction

Lumbar spinal stenosis represents a significant cause of pain and disability in the aging population. Compression of the neural elements occurs with changes in the local anatomy. Many studies suggest that the ligamentum flavum is a key factor in the pathogenesis of lumbar spinal stenosis [1–6]. The degenerative cascade which includes disc deterioration and facet joint arthrosis, also leads to ligamentum flavum in-folding, hypertrophy, and fibrosis [2, 4, 7, 8]. These changes have been associated with inflammatory changes as well as increased mechanical stresses [2, 3, 5, 6, 9–12].

Even though many studies have shown the significance of ligamentum flavum hypertrophy in patients with spinal stenosis or at the advanced stage of spondylosis, few studies have systematically examined ligamentum flavum thickness and its relation to age and lumbar level at early stages of the degenerative cascade [1, 5, 13–16]. Previous studies measuring ligamentum flavum thickness have differed in their method of measurement, using either computed tomography (CT) or magnetic resonance (MR) imaging [2, 5, 13–16]. Most of these studies lack a direct comparison between patients with lumbar spinal stenosis and a control group. Furthermore, attempts to quantify the thickness of the ligamentum flavum have used single measurements, ignoring possible differences in laterality and location of stenosis, i.e. central versus lateral. More importantly, few studies exist that examine the possible correlation between ligamentum flavum thickness and other factors such as disc height and grade of disc degeneration.

Using enhanced MR images as well as bilateral medial and lateral measurements of ligamentum flavum thickness, the current study examined ligamentum flavum thickness across different age groups from 20–60 years of age, gender, and lumbar level in individuals with and without low back pain symptoms. In addition, the effects of disc height and grade of disc degeneration on ligamentum flavum thickness were also analyzed.

## Materials and Methods

### Ethics Statement

A total of 63 volunteers were enrolled in this study (Rush University Medical Center IRB Approval No. 00042801; study no. ORA L05090801) after providing written informed consent. The IRB-approved consent documents were signed both by the principal investigator and the subject and a copy was provided to the subjects. Study L05090801 is a larger study that probed the relationships between disc and facet degeneration and in vivo lumbar kinematics, which involved lumbar spine imaging (both CT and MR) of subjects in various torso positions (supine and axial rotation, as shown elsewhere[14, 17–19]). One of the imaging modalities included in the study was MRI to evaluate the quality of the subjects’ intervertebral discs. Since the field of view of the MRI data also includes the ligamentum flavum, it provided the authors with the necessary data for the analysis presented here.

### Subject Inclusion/Exclusion Criteria

Each subject was screened by the authors for pre-existing lumbar spine pathology and pain episodes in order to classify each subject as asymptomatic or symptomatic. Exclusion criteria for the asymptomatic group were ongoing low back pain, previous spinal surgery, history of low back pain, age over 60 years, obesity, or claustrophobia or other contraindication to MR or CT imaging. Inclusion criteria for the low back pain group were recurrent pain in the low back with at least two episodes each lasting at least 6 weeks and brought on by modest physical exertion. Reasons for exclusion in the low back pain group were prior surgery for back pain, age over 60 years, claustrophobia or other contraindication to MR and CT imaging, severe osteoporosis, severe disc collapse at multiple levels, severe spinal stenosis, destructive process involving the spine, litigation or compensation proceedings, extreme obesity, congenital spine defect, or previous spinal injury.

### Image Data Acquisition and Post-processing

**Fig 1:**
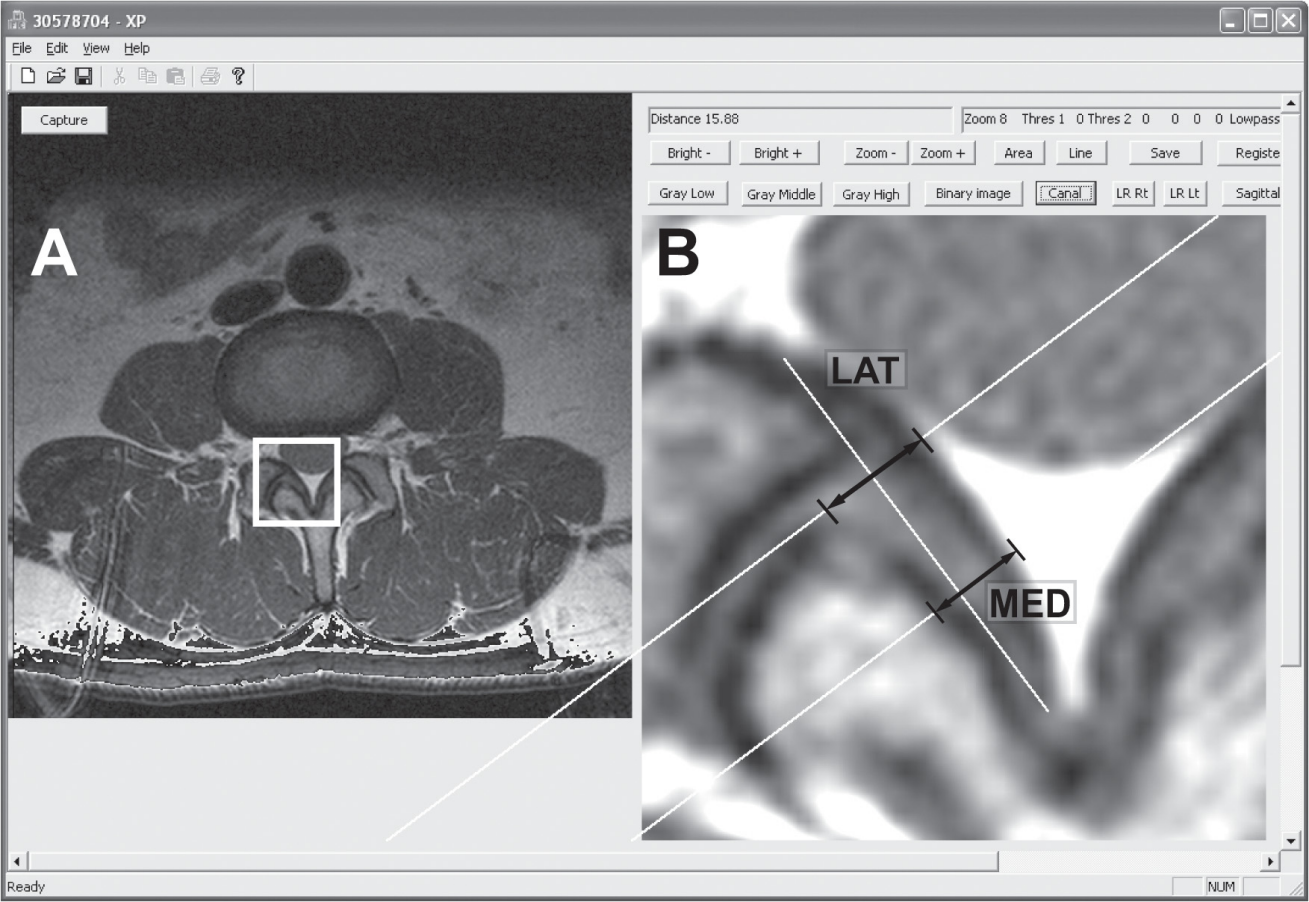
Screen capture of the image analysis program interface. (A) An original PD MR image. The region of interest box (shown as a white square) is selected for analysis. (B) Bilinear interpolation result showing an 800% enlarged image. Arrows indicate lateral thickness (LAT) and medial (MED) thickness.

Magnetic resonance imaging was used to provide a cross-sectional view of the lumbar spinal canal from L1/2 to L5/S1. A 1.5-T MR (GE Excite 2, GE Healthcare, Milwaukee, WI) unit was used to obtain 3.0 mm-thick axial proton-density images using standard clinical protocol in which the slice orientation is parallel to the endplates at each disc level. The center slice (at mid-disc level) was selected for subsequent analysis from the axial images at each lumbar level from L1/2 to L5/S1. After selection, the MR images were analyzed by custom software written in Visual C++ (Microsoft Foundation Class). Ligamentum flavum images were captured and enlarged 800% using a bilinear interpolation size conversion algorithm that allowed for enhanced image quality and provided a basis for more accurate measurement [17]. Ligamentum flavum thickness was assessed using bilateral medial and lateral measurements. To determine medial and lateral measurement locations, distances were measured from the lateral border of the ligamentum flavum to midline to define the length; with the medial measurement set at on-third of the length from midline, and the lateral measurement set at two-thirds of the length from midline. Measurement lines were perpendicular to ligamentum flavum length (Fig 1). Ligamentum flavum thickness was compared between right and left sides, and the data was pooled to form average thickness values. Average thickness by medial and lateral methods was examined by levels (L1-L5), age groups (20’s, 30’s, 40’s, 50’s), gender, and symptomatic versus asymptomatic. Disc height at each level was measured by the least-distance measurement method in three-dimensional models created by CT images taken for the same subject [14, 20]. Disc grade was assessed on the mid-sagittal T2 MRI scan using the Pfirrmann classification [21]. Ligamentum flavum thickness data were then compared to disc height and disc grade (Pfirrmann classification) to assess for possible correlations.

### Statistical Analysis

Paired *t*-tests were used to compare thickness on left and right sides. Unpaired *t*-tests were used to compare measurements between genders and asymptomatic/symptomatic subjects. Differences based on age, spinal level and disc degeneration grade were analyzed using a one-way ANOVA with a Fischer’s *post-hoc* test. Correlation between the ligamentum flavum thickness and disc height was evaluated using linear regression analysis and Pearson’s correlation coefficients. Statistical significance was set at the level of *p* < 0.05. Results are presented as mean and standard deviation.

## Results

### Lumbar Levels

A total of 63 individuals were studied, with 36 in the asymptomatic group, and 27 in the symptomatic group (mean age 31.9 ± 8.6 years and 41.2 ± 10.1 years, respectively). Demographic data are summarized in Table 1. In comparison of right versus left-sided ligamentum flavum thickness, differences were not significant by medial or lateral measures at any level. Right and left-sided thickness measures were compiled for average thickness, and are summarized in Table 2. When examined by lumbar level, the overall trend was an increase in ligamentum flavum thickness by level caudally from L1/2 (labeled L1) to L4/5 (labeled L4). With the medial measurement, thickness was smallest at L1 and L5, and increased caudally from L2 to L4; statistical significance was found between all levels except L1 and L5 (*p* = 0.13). Using the lateral measurement, the order of ligamentum flavum thickness from least to greatest was L1, L2, L5, L3, and L4. Statistical significance was achieved at fewer levels than with the medial ligamentum flavum measure, with L1 thickness being significantly less than L3, L4, and L5; and L2 significantly less than L4. At all levels except L5/S1, medial ligamentum flavum thickness was greater than lateral ligamentum flavum thickness.

**Table 1:** Demographic distribution among asymptomatic and low back pain patients. Percentages are calculated with respect to the total number of subjects.

	Asymptomatic	Low Back Pain Patients
Total Number	36	27
Males	23 (36.5%)	13 (20.6%)
Females	13 (20.6%)	14 (22.2%)
Age: 20s	17 (27.0%)	3 (4.8%)
Age: 30s	13 (20.6%)	14 (22.2%)
Age: 40s	4 (6.3%)	6 (9.5%)
Age: 50s	2 (3.2%)	7 (11.1%)

**Table 2:** Mean (SD) thickness values by age group.

	Medial Thickness (mm)		Lateral Thickness (mm)	
Age Group	Mean	SD	Mean	SD
20's	3.08	0.83	2.77	0.57
30's	3.28	0.88	3.05	0.70
40's	3.39	0.84	3.10	0.55
50's	3.54	0.71	3.32	0.65

### Gender

Average medial thickness of male subjects (3.30 ± 0.78 mm) was not significantly different from that of females (3.23 ± 0.93 mm; *p* = .5042). Similar results were obtained when comparing lateral thickness in males versus females (3.06 ± 0.66 mm versus 2.93 ± 0.64 mm, respectively, *p* = 0.078). This statistical trend was confirmed when the gender effects were further examined by level, a similar pattern emerged as male ligamentum flavum thickness was greater than female ligamentum flavum thickness, but without statistical significance: *p* = 0.4475 for the medial thickness and *p* = 0.0673 for the lateral thickness, respectively).

### Age Groups

Medial and lateral ligamentum flavum thickness increased by age group (Table 2). Statistical significance varied by measurement method. For the medial measurement, the youngest group was significantly smaller than the two older groups (40s and 50s, *p* < 0.03), whereas the lateral measurement showed more differences, as follows: 20s<30s, 20s<40s, 20s<50s and 30s <50s, *p* < 0.0185. Age group differences were not subdivided by level due to the small sample size of patients 50 years and older.

### Low Back Pain Symptoms

Results for the low back pain group versus the asymptomatic group are summarized in Table 3. Overall, greater medial thickness was found in symptomatic subjects (3.41 ± 0.82 mm) than in asymptomatic subjects (3.16 ± 0.85 mm; *p* = 0.0098). Lateral thickness in symptomatic patients (3.14 ± 0.57 mm) was also shown to be significantly greater than that in asymptomatic subjects (2.91 ± 0.70 mm), with *p* = 0.0019.

**Table 3:** Mean (SD) thickness among asymptomatic and low back pain patients.

	Medial Thickness (mm)				Lateral Thickness (mm)			
Spinal Level	Asymptomatic		Low Back Pain		Asymptomatic		Low Back Pain	
	Mean	SD	Mean	SD	Mean	SD	Mean	SD
Overall	3.16	0.85	3.41	0.82	2.91	0.70	3.14	0.57
L1	2.79	0.55	3.03	0.52	2.52	0.51	2.92	0.44
L2	3.13	0.59	3.45	0.74	2.77	0.57	2.99	0.49
L3	3.37	0.80	3.79	0.80	2.96	0.52	3.38	0.56
L4	3.85	0.79	3.92	0.92	3.13	0.67	3.27	0.67
L5	2.66	0.94	2.863	0.53	3.16	0.96	3.12	0.55

**Fig 2:**
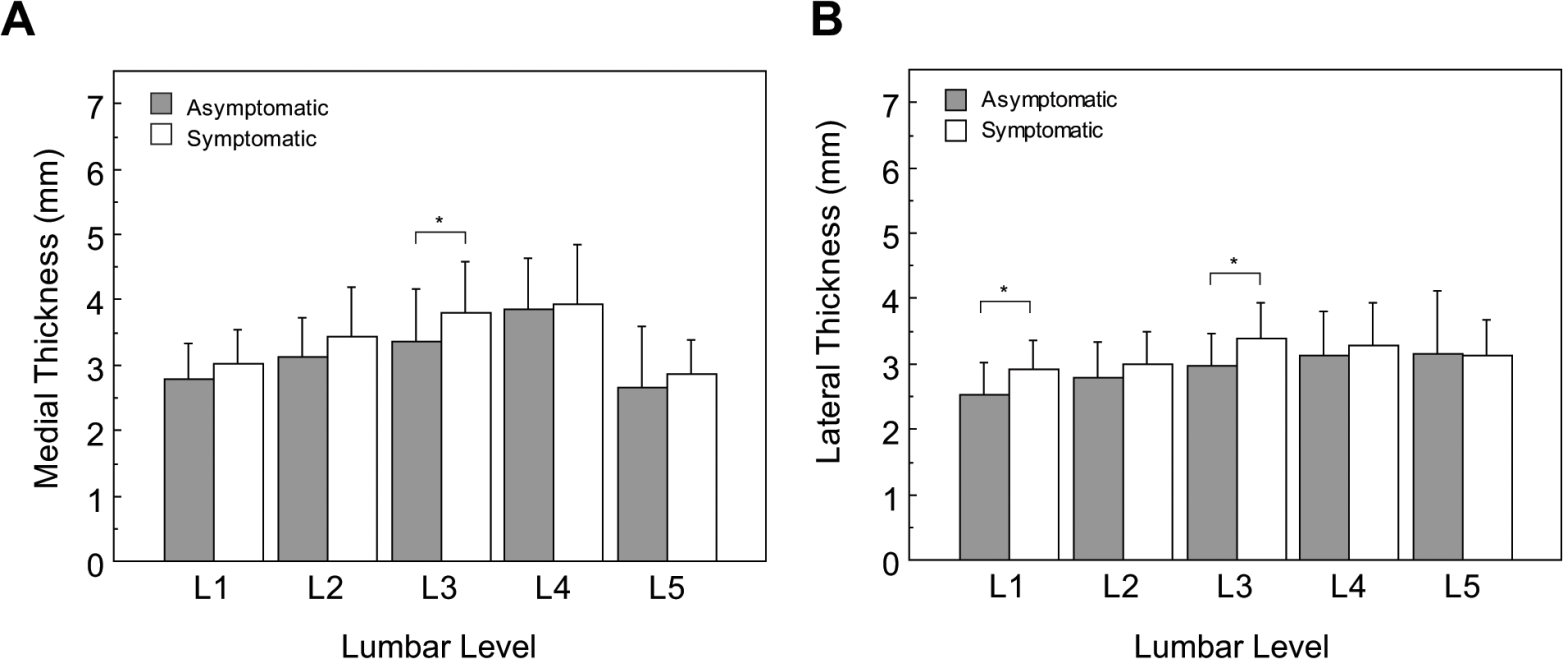
Medial (A) and Lateral (B) ligamentum flavum thickness variation by lumbar level. Comparison between asymptomatic and chronic low back pain subjects segregated by spinal level. Error bars span one standard deviation. Asterisks denote significant differences with * *p* < 0.05. In (A) the symptomatic medial thickness tended to be larger than in the normal (L1: p = 0.0839, and L2: p = 0.0652, respectively). All other comparisons did not reach significance.

Thickness differences between low back pain and asymptomatic subjects were then examined by level. It was found that low back pain subjects had slightly thicker medial ligamentum flavum in the upper lumbar all levels, except at L4 and L5 where thickness was almost equal and no significant differences were found (*p* = 0.74 and *p* = 0.32, respectively). These differences were not significant at L1 and L2 (*p* = 0.08 and *p* = 0.06, respectively), however only L3 reached significance at *p* = 0.04 (Fig 2A). Using the lateral measurement, low back pain subjects had thicker ligamentum flavum at L1 (2.92 ± 0.44 mm) and L3 (3.38 ± 0.56 mm) compared to asymptomatic subjects at L1 (2.52 ± 0.51 mm) and L3 (2.96 ± 0.52 mm), respectively. These differences reached statistical significance (*p* = 0.0018 and *p* < 0.0031, respectively, Fig 2B).

### Disc Height, Disc Grade

**Fig 3:**
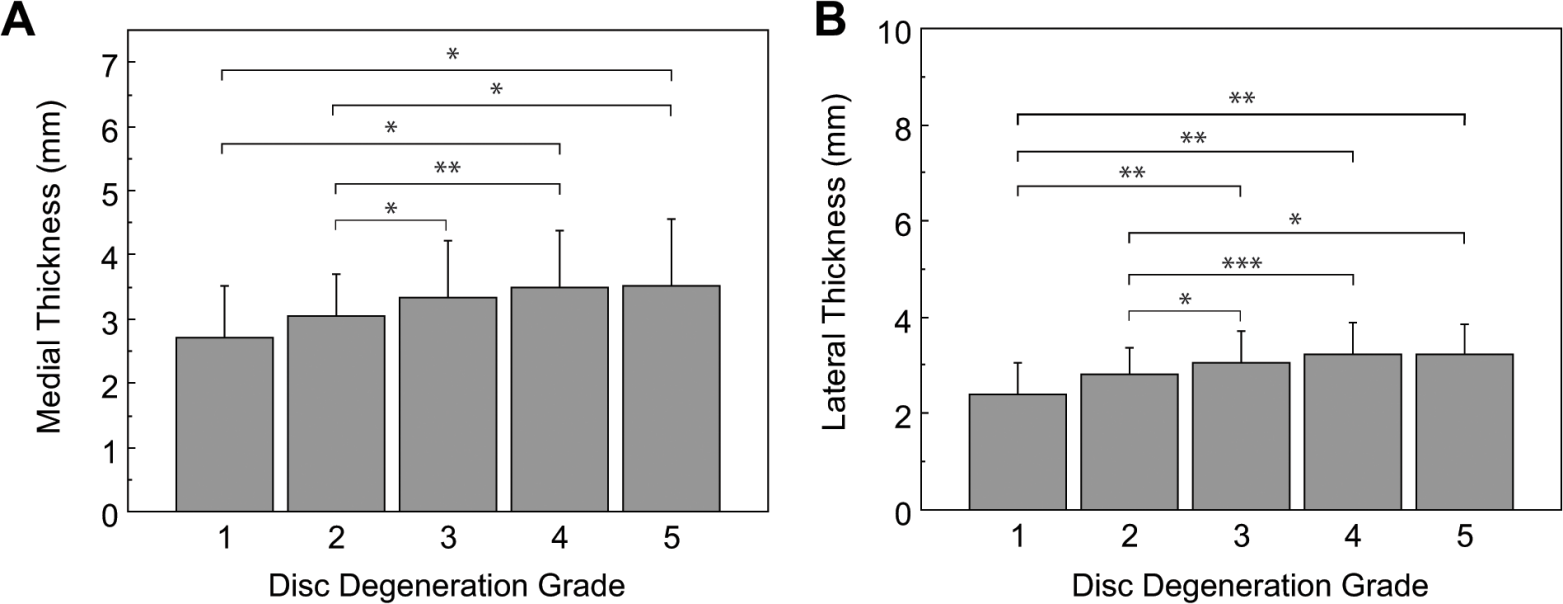
Ligamentum flavum thicknessby disc grade. Error bars span one standard deviation. (A) Medial: Asterisks denote significant differences as follows: * *p* < 0.05; and ** *p* < 0.001. Medial thickness in grade 3 tended to be larger than that in grade 1 (p = 0.0601). (B) Lateral: Asterisks denote significant differences as follows: * *p* < 0.05; ** *p* < 0.01, and *** *p* < 0.0001. Lateral thickness in grade 2 tended to be larger than that in grade 1 (p = 0.096). All other comparisons did not reach significance.

Medial and lateral ligamentum flavum measurements were related to disc height, and disc grade. Two-tailed Pearson correlation coefficients were calculated for the relationships between disc height and both medial and lateral ligamentum flavum thickness at each lumbar level, showing low but significant (r = 0.229, *p* < 0.01) correlation between medial thickness and disc height, and an even lower relationship between lateral thickness and disc height (r = 0.104, *p* = 0.067) that was not significant, but a trend nonetheless. It is worth noting that between both thickness measurements, a moderate correlation coefficient was obtained (r = 0.418. p < 0.01). Thickness measurements are associated with disc degeneration grade as shown in Fig 3.

## Discussion

Using MR imaging and custom-developed, spine specific measurement techniques, we examined the thickness of the ligamentum flavum in asymptomatic and chronic low back pain individuals. In our series of 63 subjects, we found ligamentum flavum thickness differs between symptomatic and asymptomatic subjects and increases with higher disc degeneration grade, older age and lower lumbar level.

Ligamentum flavum thickness was measured at two different locations in the present study. Previous studies have not agreed upon a uniform method of measuring ligamentum flavum thickness. While certain studies have utilized an area averaging technique, others have assessed ligamentum flavum thickness using a single measurement located at the approximate “middle” of the ligamentum flavum [2, 5, 13, 15, 16]. We believe the medial and lateral measurements provide a better understanding of how ligamentum flavum thickness varies and may affect symptomatology. Lateral ligamentum flavum thickness was significantly greater in low back pain subjects compared to asymptomatic subjects. Medial ligamentum flavum thickness was also greater in low back pain subjects, but the difference was not statistically significant. The finding that an increase in lateral ligamentum flavum thickness was more significant than medial ligamentum flavum thickness is also notable. This is reasonable, given the close proximity of the lateral measurement to the facet joint, where motion and instability are most appreciable. Lateral recess stenosis, which is a frequent finding in older patients with lumbar radiculopathy, may have an earlier basis with lateral thickening of the ligamentum flavum and low back pain. Additionally, both measurements were moderately correlated, which was an expected result. These findings of our study, together with the current literature, support the idea that early changes in the ligamentum flavum may play a key role in chronic low back pain symptoms.

While traditionally the focus of discussion has been on the bulging of the disc leading to compression of the cauda equina, there is growing evidence that identifies ligamentum flavum buckling and hypertrophy as the key pathologic feature of spinal stenosis. Hansson *et al*. examined 24 individuals using MRI before and after an external axial load. The authors found the ligamentum flavum to be the primary cause of canal encroachment, with bulging of the ligament leading to 50 to 85% of the reduction in canal area [22]. Since axial loading causes disc height loss [23], this study probed any possible correlations between disc height and the ligamentum flavum thickness. However, no correlation was found between the disc height and the ligamentum flavum thickness in our series. Given that disc height loss associated with disc degeneration is a slow process, bulging of the ligamentum flavum may not occur under a gradual loss of disc height due to the elastic nature of the ligamentum flavum.

In contrast, the present study found a low, positive but significant correlation between ligamentum flavum thickness and *disc grade* (using the Pfirrmann classification system). The fact that moderate correlation coefficients were obtained between thickness measurements and disc height also shows that loss of disc height might be one of the contributing factors to instability associated with disc degeneration [18, 19] and stenosis. Contribution of mechanical stresses to ligamentum flavum thickening has been reported in the literature. Sairyo et al. [5] used a multidisciplinary approach to study the pathomechanism of ligamentum hypertrophy and found transforming growth factor-beta to be related to the stimulation of fibrosis and summarized the process of ligamentum flavum hypertrophy as beginning with mechanical stress inducing tissue damage, leading to inflammation and scarring, and finally fibrosis. Histologic studies have identified inflammatory markers including tissue inhibitors of matrix metalloproteinases [11, 24], microRNA molecules such as miR-155 [25] and increases in connective tissue growth factor [26] to be related to ligamentum flavum hypertrophy and fibrosis. Although pathogenesis of inflammation in the ligamentum flavum appears to be multifactorial, abnormal movements of the motion segment could cause mechanical stress and, in tern, inflammatory reaction.

The limitations of our study may stem from the relatively small sample size and availability of MRI image data. A *post hoc* analysis was completed to reveal a power of (1-ß) = 0.87. However, it is still possible that this report may be underpowered to reveal differences that truly exist. The selection criteria for the low back pain group excluded subjects with severe disc collapse and stenosis. Although this serves to increase the specificity of our findings, the sensitivity of the study may have been compromised. Given that the parent study for this research was not designed to obtain normative data, our results might be limited due to relatively small subject population. However, one of the intended contributions to the field via this report, is communicating the development and use of this new method to quantify the thickness of the ligamentum flavum using clinically-available image sets. Future studies including individuals older than 60 years of age will shed light on the degenerative cascade of the spondylosis including ligamentum flavum hypertrophy.

With respect to the choice for the mid-image slice, alas, 3.0 mm is still thicker than ideal, but that comes also with the fact that it is practically the only slice available that captures the ligamentum flavum at mid disc. It is also, difficult to standardize and adjacent slices might not be consistent in terms of displaying the appropriate views, especially in the posterior processes. The scout view helped in choosing this image to account for orientation and location.

## Conclusions

Direct relationships between increasing age, ligamentum flavum thickness and have been insinuated by others working in the field of spine biomechanics. Our study confirmed an increase in ligamentum flavum thickness both medially and laterally as age increased and as lumbar level increased (caudally), through the L4/5 level. The thickness at the L5/S1 level varied by method but was comparatively thicker using the lateral measurement. This finding may represent the degenerative process in the ligamentum flavum that occurs with age and also supports the reactive nature of the ligamentum flavum to increased mechanical forces at the lower lumbar levels.
